# Relabeling for Indoor Localization Using Stationary Beacons in Nursing Care Facilities

**DOI:** 10.3390/s24020319

**Published:** 2024-01-05

**Authors:** Christina Garcia, Sozo Inoue

**Affiliations:** Graduate School of Life Science and Systems Engineering, Kyushu Institute of Technology, 2-4 Hibikino, Wakamatsu Ward, Kitakyushu 808-0135, Japan; sozo@brain.kyutech.ac.jp

**Keywords:** oversampling, data augmentation, machine learning, signal measurement, signal pattern, relabeling, indoor localization, beacon, nursing care

## Abstract

In this study, we propose an augmentation method for machine learning based on relabeling data in caregiving and nursing staff indoor localization with Bluetooth Low Energy (BLE) technology. Indoor localization is used to monitor staff-to-patient assistance in caregiving and to gain insights into workload management. However, improving accuracy is challenging when there is a limited amount of data available for training. In this paper, we propose a data augmentation method to reuse the Received Signal Strength (RSS) from different beacons by relabeling to the locations with less samples, resolving data imbalance. Standard deviation and Kullback–Leibler divergence between minority and majority classes are used to measure signal pattern to find matching beacons to relabel. By matching beacons between classes, two variations of relabeling are implemented, specifically full and partial matching. The performance is evaluated using the real-world dataset we collected for five days in a nursing care facility installed with 25 BLE beacons. A Random Forest model is utilized for location recognition, and performance is compared using the weighted F1-score to account for class imbalance. By increasing the beacon data with our proposed relabeling method for data augmentation, we achieve a higher minority class F1-score compared to augmentation with Random Sampling, Synthetic Minority Oversampling Technique (SMOTE) and Adaptive Synthetic Sampling (ADASYN). Our proposed method utilizes collected beacon data by leveraging majority class samples. Full matching demonstrated a 6 to 8% improvement from the original baseline overall weighted F1-score.

## 1. Introduction

The increasing population of older adults is impacting the nursing workforce, leading to a shortage of skilled staff [1]. As the demand for services grows, the use of nursing homes is also escalating, resulting in a rise in the patient-to-caregiver ratio [2,3]. Research efforts to better comprehend activity patterns during patient assistance are significant to leverage staff and improve elderly care delivery [4].

Indoor Positioning System (IPS) allows localization within enclosed spaces, facilitating navigation and the tracking of individuals or objects through a network of transmitters and receivers [5,6]. Employing indoor positioning to monitor care routine and patient assistance is helpful to support nursing records and to optimize care response [7,8,9]. Several key positioning techniques exist, but the standard approach includes trilateration, triangulation, multilateration, and fingerprinting [10]. However, these techniques are limited by environmental factors such as hardware requirements, setup complexity, signal obstruction, and time synchronization.

IPS differ in technology, signal measurement, and localization techniques tailored to suit the environment and particular use-case requirements [8]. Radiofrequency-based technologies [11], specifically Wi-Fi [12,13,14,15,16], Radio Frequency Identification (RFID) [17,18], and Bluetooth Low Energy (BLE) [19,20,21], are typically preferred considering flexibility to setup and integration with IoT. Recently, studies on Wi-FI have been optimizing line of sight (LOS) and non-line of sight (NLOS) [22,23] approaches, including combination with vision [24] for localization. Furthermore, sensors such as IMU [25] and Geomagnetism [26] are integrated in movement and positioning. However, considering restrictive data collection in caregiving activities, beacons are preferred in nursing homes with straightforward installation and lesser complexity imposed on participants [3,27]. The presence of unavoidable equipment in the facility can also hamper the collection of geomagnetic data. Given that Indoor Positioning Systems (IPS) are contingent upon the specific use case and environment, Bluetooth Low Energy (BLE) has been selected based on target availability, straightforward deployment, and privacy considerations pertinent to elderly individuals. Additionally, the energy-efficient power consumption of BLE allows longer data collection with minimal disturbance in the facility monitoring the device remotely.

A common challenge to indoor localization accuracy is low quality of signals. Outlier detection [28] and filtering methods are applied to resolve this issue. Moving average [29], weighted average [30], Bayesian sequential Monte Carlo [20], and Gaussian filtering [31] has been proposed for signal smoothing to achieve better positioning. Kalman filter [21,32] is a widely used method for filtering signals in IPS. In addition, machine learning [7,10,14,29,33] and deep learning [12,30,34] methods are being employed to optimize signal features which are not covered by standard localization techniques. Network issues, environmental factors affecting signal quality, and hardware malfunction can impact collected data. Moreover, data imbalance due to unequal representation of different areas or activities within the facility in a real-world setting affects indoor positioning accuracy [2].

Data augmentation methods are utilized to address the issue of class imbalance, including in IPS applications. Data augmentation is a method to artificially increase the training dataset by creating a modified version of the current data. To increase real data, Random Sampling (RS) [17] is commonly applied to minority class samples by duplication. On the other hand, creating synthetic samples is an alternative method. Adaptive Synthetic Sampling (ADASYN) [35] and Synthetic Minority Over-sampling Technique (SMOTE) [36] both generate synthetic data points for the minority class by interpolation based on the nearest neighbors principle. Currently, existing oversampling techniques suffer from repeating data, resulting in overfitting, while synthetic samples are prone to noise and data misrepresentation. Since augmented data are intended to reflect actual samples, oversampling should be carefully considered. At present, augmentation methods do not optimize real data from the majority classes, and few studies exist that investigate the use of signal patterns for augmenting training data in the context of indoor localization [2].

This paper introduces a data augmentation approach to address the challenge of unequally represented locations in beacon-based indoor localization. By analyzing signal patterns in different rooms, we successfully implement a relabeling strategy, utilizing Received Signal Strength Indicator (RSSI) values from one location as a proxy in another. As more nursing homes adopt IPS with IoT, this research aims to leverage beacon data collected in real-world environments to improve indoor localization. The main objective of this research is to develop an augmentation method by increasing training samples with beacon data from different rooms to improve location detection of minority classes. We focus on identifying semantic locations where rooms are preferred over exact geographical positions to support caregiving records. Specifically, this study aims to address the following research questions:

1. How do we identify and match signal patterns between different locations in the facility? We utilize standard deviation and Kullback–Leibler divergence to analyze signal patterns, facilitating the identification of similar beacons across minority and majority classes.
2. How can we use the samples from other beacons to augment location with less data? We propose a novel relabeling approach that reassigns signals from matching beacons to areas with fewer samples, thereby addressing data imbalance issues.
3. What is the performance of oversampling based on signal pattern relabeling compared to existing augmentation methods? To evaluate, we compare the performance of relabeling with Random Sampling, ADASYN, and SMOTE applied to our collected real-world data from a nursing facility. To further assess, we test relabeling to different rooms to validate its effectiveness.

We employ different augmentation techniques in the training set comprising 3.5 days of data and perform indoor localization using 1.5 days of test data. Overall and target class F1-scores are measured to account for the data imbalance, including precision, and recall. Our proposed method achieves an improved target class F1-score of 27% to 40% compared to the baseline data. With full matching, the relabeling method demonstrates a 6 to 8% improvement from the original baseline overall weighted F1-score. KL divergence results in a better F1-score than the standard deviation for both full and partial matching. This method effectively expands the training set, enhancing model accuracy, as demonstrated in a nursing care facility where beacon devices and mobile applications are employed for data collection.

The subsequent sections of this paper are structured as follows: Section 2 covers the relevant research on indoor localization with BLE technology, including the distinction of our work over other localization and oversampling techniques. In Section 3, we elaborate on the proposed method, which encompasses signal pattern and relabeling approach, while Section 4 covers the data collection process, including the evaluation of proposed relabeling in comparison to baseline and other augmentation strategies. A comprehensive analysis of the results follows in Section 5 emphasizing the significant insights from our research. Section 6 concludes the paper, highlighting the main contributions and outlining potential directions of future work.

## 2. Related Literature

In this chapter, we cover relevant works on the design of indoor positioning implemented in the nursing facility. With the advent of the Internet of Things (IoT), devices that easily connect to the network such as beacons, tags, and mobile devices [2,37,38,39,40] are preferred in system design. Signal measurements are generally based on time, angle, and received signal strength (RSS) [28,29,31,38,41].

### 2.1. Indoor Localization with Beacons

Beacons are battery-driven radio transmitters used in indoor positioning with proximity sensors that emit BLE signals [42,43]. Unlike the classic Bluetooth, which is connection-oriented, BLE has advertising functionality and does not necessarily have to pair [44,45]. Beacons are preferred in IPS since they are flexible, easy to deploy, and cost-effective, with low power consumption that can last up to a year [46].

BLE beacons are used to track nursing activities to better understand care routines, which is crucial to optimize workload distribution considering the low staff-to-patient ratio [47]. Existing systems combine beacons with Wireless Local Area Networks (WLAN) for tracking both patients and nurses by analyzing RSSI, Time of Arrival (ToA), and the Angle of Arrival (AoA) [7]. This involves the setup of more hardware as receiver and transmitter modules. BLE beacons are often paired with smartphones used by caregivers for detection. To automatically record daily caregiving routines, beacons are used for time-spatial recognition [5,9].

In some nursing facilities, multiple patients share a common room. Considering the effects of setting up indoor positioning on the privacy of the elderly, which can affect their social interaction, the placement of beacons should be carefully considered in planning IPS [27]. Studies on nursing homes commonly use RSS and BLE for indoor localization [3,9,27] with its energy-efficient power consumption. However, noise and multipath can affect the signal quality and positioning performance.

### 2.2. Data Augmentation

The uneven distribution of classes within a dataset is referred to as data imbalance, which is a prevalent problem in machine learning. This indicates that the classifier performs well for the majority class but poorly for the minority class. Since minority classes are crucial to solving many real-world issues, correctly categorizing samples from this class is similarly crucial [48,49,50].

Data augmentation is significant in improving indoor positioning systems. The technical and non-technical challenges of IPS in real-world environments [51] have impacted the precision of indoor positioning with uncontrolled variables such as signal interference, physical obstructions, and hardware breakdown. Data collection in on-site scenario [52] affects data quality and labeling, resulting in an imbalanced and unequal representation of classes in dataset [2,53]. In order to create a robust and unbiased model for IPS, data augmentation shows up as a crucial and imperative approach to address data imbalance. By applying several changes to the current dataset, data augmentation creates synthetic data that diversify the training set and lessens the effects of class imbalance [54,55,56].

Three basic approaches dominate data-level solutions to the class imbalance issue: Random Sampling [57,58], Synthetic Minority Over-Sampling Technique (SMOTE) [56], and Adaptive Synthetic Sampling (ADASYN) [35]. By randomly adding additional copies of selected minority classes to the training data, Random Over-Sampling balances class distribution [57]. On the other hand, instead of oversampling by replacement, ADASYN and SMOTE oversample the minority class by producing synthetic instances [36]. However, these techniques have several limitations.

Overfitting can result from Random Over-Sampling since duplicates of minority-class samples are added to the dataset without adding new information. If the original data are already highly dimensional, this increases the calculation cost and lengthen the classifier’s training period. Conversely, Random Under-Sampling randomly removes instances from the majority class, potentially leading to the neglect of essential data.

When training a model, applying SMOTE creates a linear mapping of the data, which can lead to overfitting issues. Also, there is a risk of overlap because the SMOTE method does not account for the position of general data close to the uncommon class data [49,56,59,60] Similarly with ADASYN, possible class overlap occurs in boundary areas as oversampling targets to resample between neighboring minority and majority classes. In general, both synthetic approaches are sensitive to noise and need parameter tuning as both are dependent on data distribution [61].

A combination of augmentation techniques has been applied in indoor localization [62,63] including adding information to the reference dataset and deep learning-based approach [34,64], yet these involve computational complexities. Few studies have focused on using methods based on signal patterns between sensors to augment training data specifically for indoor localization purposes in nursing homes.

Accurate labels ensure that models learn the correct patterns and relationships in the data. In machine learning, relabeling is employed in the data augmentation process to handle proper class labeling, which is crucial for the accuracy of supervised learning models. In existing studies, relabeling is commonly applied to preserve the original class labels [65]. Recently, relabeling has been used to address class imbalance when using logistic regression by assigning new labels to classes with fewer instances [66]. Data augmentation leading to the loss of label information can reduce model performance.

### 2.3. Signal Pattern

Signal patterns refer to the specific way that radio frequencies, like Bluetooth, behave in an indoor setting. Fluctuations in the signal are essential metrics providing valuable insights into the alterations of signal intensity that occur within indoor settings [67]. Trilateration and triangulation are traditional localization techniques that utilize geometric principles to assess signal behavior, measuring distance and angle from reference points.

In the non-conventional approach, statistical techniques are optimized to handle inherent variability and uncertainty in the signal-based analysis. The received signal’s standard deviation reflects the signal strength fluctuations at different positions or distances within an indoor environment [68,69,70]. On the other hand, variance measures the spread of the RSSI values around the mean, offering perspectives on the consistency of the signal strength measurements at different locations [68]. KL divergence is a measure of the difference between two probability density functions. In IPS, it is calculated to measure similarity in the online phase of unlabeled data in existing database and up-to-date data [10].

Integrating multiple signal sources, sensor fusion techniques, and machine learning provides more robust and reliable localization. Implementing adaptive algorithms that can learn and adjust to environmental changes further improves positioning performance. In this study, we delve into the signal patterns between locations by employing statistical techniques, specifically standard deviation and KL divergence, to measure features to find matching patterns for oversampling.

In this work, we propose to compare the signal pattern features of labeled beacon data from different locations and determine the divergence between minority and majority classes as a foundation for data augmentation. By considering the layout of the care facility, we focus on leveraging the current data from stationary beacons by using the majority class to oversample locations with less BLE data. We utilize relabeling to update labels of the augmented data derived from other locations, aligning them with the labels of the minority class. Specifically, signal patterns observed from selected positioned beacons from various rooms are utilized to guide the relabeling process. Full and partial matching represent two distinct relabeling variations that take into account the comprehensive arrangement of installed beacons.

This research strategically deploys BLE beacons to enable indoor localization of nursing staff, ensuring that the architecture of the nursing home remains unaltered and that caregiving services are delivered without interruption. This work prioritizes identifying semantic room locations over exact coordinates, considering the relevance to caregiving records. To protect the privacy of patients, all devices are mounted outside the doors of patient rooms.

## 3. Material and Methods

**Figure 1:**
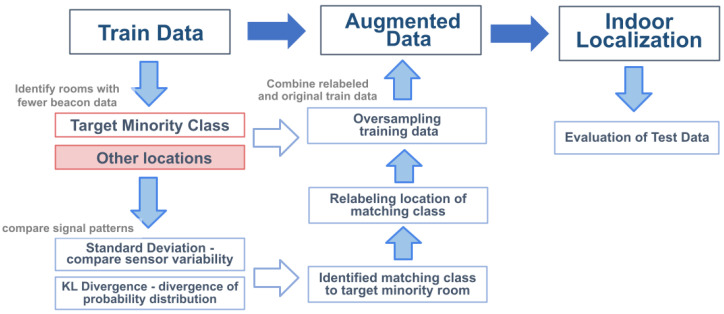
Proposed oversampling approach based on signal pattern relabeling for indoor localization.

This section introduces our developed relabeling method depicted in Figure 1 for beacon data augmentation.

In this approach, we aim to increase the training data sample, particularly locations with less beacon data. Initially, training data are analyzed to identify rooms with fewer beacon signals, termed as the ”Target Minority Class”. To oversample, we identify matching locations to minority classes from other locations by comparing signal patterns using standard deviation and KL divergence. To proceed with data augmentation, we apply relabeling to the identified matching class by updating the location label similar to the target minority. The relabeled set is then added to the original training data, increasing the set. The augmented data are then used to enhance the indoor localization employing machine learning, with the model’s efficacy evaluated using test data.

**Figure 2:**
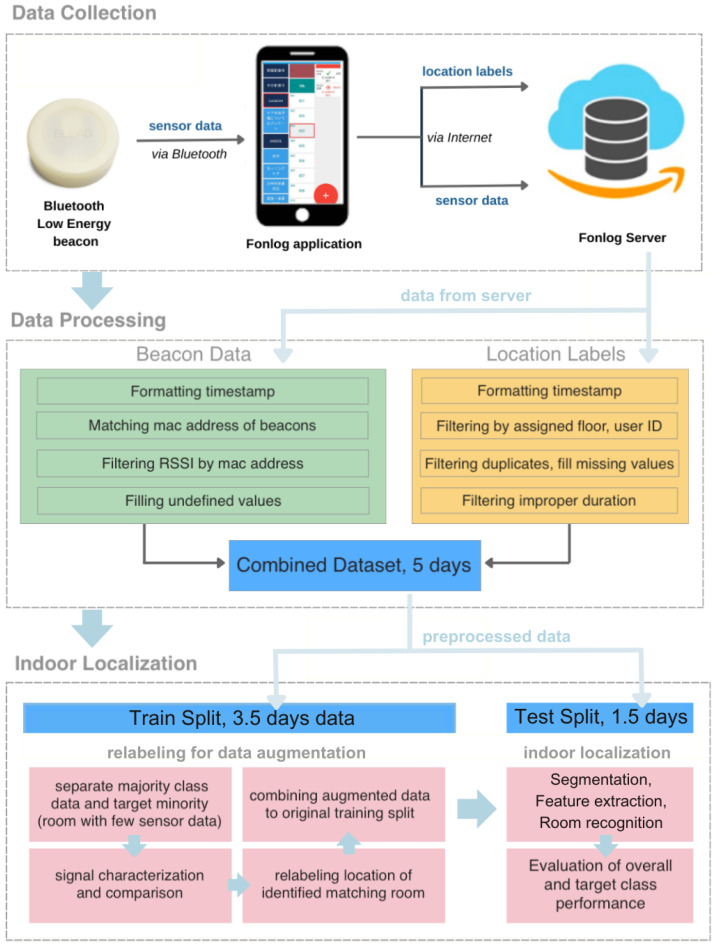
An overview of the proposed indoor positioning system in nursing care facility.

The subsection commences with information on the beacon data from the site. Understanding the signals detected from beacons to comprehend variability is necessary to execute relabeling. Figure 2 describes the overview of the indoor localization employed in the nursing facility from data collection to the use of the relabeling approach.

### 3.1. Signal from Detected Beacons

**Figure 3:**
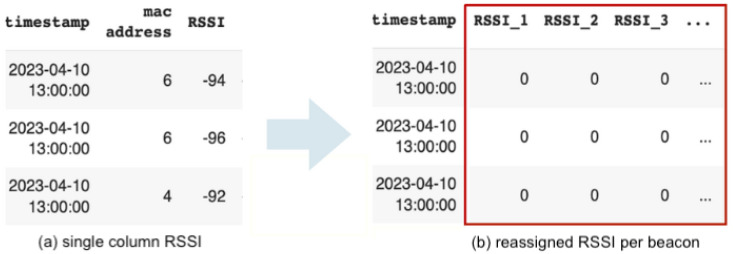
Matrix of the received signal strength from beacons. (**a**) Single column RSSI. (**b**) The corresponding RSSI for each beacon, repositioned into separate columns.

Prior to deployment, the respective MAC addresses of the beacons are recorded to match the raw data from the server, as shown in Table 1. After pre-processing the raw data, the MAC addresses of detected devices are filtered to match the beacons. The final dataframe of RSSI values is shown in Figure 3.

Based on Figure 3, we can suppose $RSSI_{t}$ represents the detected RSSI at timestamp *t* at location *m*. We then define the RSSI measurement for all beacons *n*, and the corresponding location label as in Equation (1). Overall, the signal database can be expressed as Equation (2) and the labels as Equation (3).

(1) $$RSSI_{t}=\left[RSSI_{t1},\cdots ,RSSI_{1n}\right],label_{t}=m_{t},$$

(2) $$RSSI=\{\left[RSSI_{11},\cdots ,RSSI_{1n}\right],\cdots ,\left[RSSI_{t1},\cdots ,RSSI_{tn}\right]\},$$

(3) $$label=\{m_{1},m_{2},m_{3},\cdots ,m_{t}\},$$

where *t* represents the timestamp, *n* denotes the total number of beacons installed on site, and *m* refers to the associated elderly room. With location label *m*, training data are observed to identify rooms with fewer detected signals.

### 3.2. Matching

**Figure 4:**
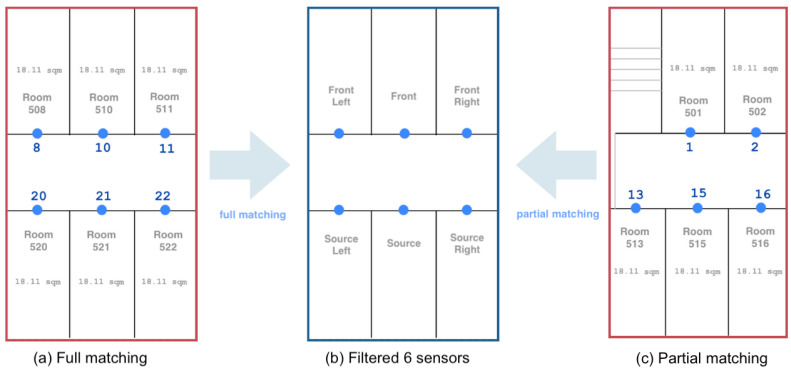
Full and Partial Matching based on selected sensors. (**a**) Full matching with complete 6 sensors. (**b**) A total of 6 sensors surrounding the location. (**c**) Partial matching with incomplete 6 sensors.

Generally, the accuracy of IPS decreases as the distance between the transmitter and receiver increases [71]. Observing this from the histogram of detected beacons and considering the setting of installed devices covering up to a 5 m range, we limit the analysis to six beacons to better comprehend signal patterns between rooms. Specifically, we only focus on elderly rooms installed with stationary beacons following a similar layout. Figure 4 shows the targeted surrounding beacons investigated for each room.

Prior to calculating the signal pattern feature, we classify rooms based on the completeness of the selected surrounding beacons. For each location, six BLE transmitters are filtered such that

(4) $$f_{m}=\left[fl_{m},sl_{m},s_{m},f_{m},sr_{m},fr_{m}\right].$$

In Equation (4), *s* is the beacon on the location (source) room, *f* is the beacon on the room in front, fl and sl are the beacons on the left side rooms while sr and fr are beacons on the right side, respectively. In full matching, we only consider elderly rooms forming complete six beacons as candidate match to the minority class. On the other hand, with partial matching, all elderly rooms are considered as possible match to the minority class regardless of incomplete sensors forming the targeted six. As an example, in Figure 4, Room 515 is considered only in partial matching where $f_{515}$ = [null,13,15,1,16,2]. Room 521, on the other hand, is also a candidate in full matching, where $f_{521}$ = [8,20,21,10,22,11]. For locations with incomplete beacons resulting in null values representing no signal detected, we replace null with zeroes for the model to better differentiate from distant beacons with higher negative values and to calculate the signal pattern feature.

### 3.3. Relabeling Based on Signal Pattern

**Figure 5:**
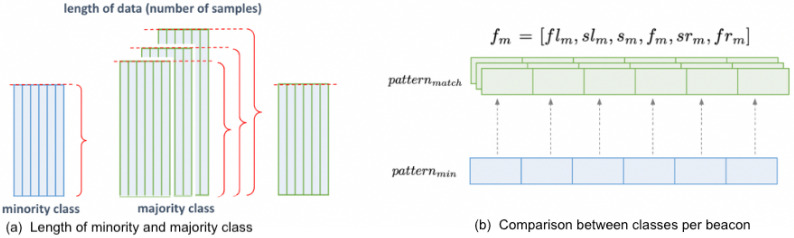
Comparing signal pattern feature between minority and majority class. (**a**) Length of samples considered. (**b**) Comparison between rooms per beacon.

A signal pattern feature is computed from the six beacons to define the signal variability in different rooms. A sub-dataframe $pattern_{m}$ containing the RSSI values of the beacons in the list $f_{m}$ is created for each room in the majority class. The sub-dataframe of the minority class is denoted as $pattern_{min}$. From these sub-dataframes, standard deviation and KL divergence are measured to represent each room’s signal pattern feature, which is then used to identify similar locations. The objective is to pinpoint the room that has a matching signal pattern with the minority class in order to move forward with the relabeling process. Initially, the majority class is downsampled to match the number of samples in the minority class as depicted in Figure 5. Outlined in Steps 1 to 4 are the procedures to calculate the signal pattern feature of $pattern_{m}$ and $pattern_{min}$. See Algorithm 1.

The signal pattern feature based on standard deviation calculates the absolute differences in standard deviation for each beacon between minority and majority classes. This approach considers individually the variability difference for each beacon before summing them. The matching location from the majority class is identified with the least standard deviation difference sum expressed in Equation (5),

(5) $$\Delta \sigma_{\mathrm{total}}=\sum \left|\sigma_{\min}-\sigma_{\mathrm{match}}\right|,$$

where $\Delta \sigma_{\mathrm{total}}$ represents the total difference in standard deviation values, $\sigma_{\min}$ is the standard deviation for a specific beacon in the minority subdataframe, $\sigma_{\mathrm{match}}$ is the standard deviation of each beacon in the candidate sub-dataframes $pattern_{m}$, and ∑ represents the summation symbol, indicating the sum of absolute differences across all beacons.   
**Algorithm 1:** Algorithm for comparing the signal pattern feature between rooms**Input:**m← room number$d_{\mathrm{train}}\leftarrow$ train data**Output:**$pattern_{\mathrm{match}}\leftarrow pattern_{m}$Identify rooms with small sample in train data, df = $d_{train}$.Define $pattern_{min}$ and $pattern_{m}$ following Equation (4) such that for *i* in labels, *m*$d_{i}=df\left[df\left[‘m’\right]\right]==“i”]$if *i* == “1”: $f_{1}$ = $\left[fl_{1},sl_{1},s_{1},f_{1},sr_{1},fr_{1}\right]$⋯elif *i* == “25”: $f_{25}$ = $\left[fl_{25},sl_{25},s_{25},f_{25},sr_{25},fr_{25}\right]$.Group $pattern_{m}$ accordingly as candidates for full and partial match.Calculate the signal pattern feature from $pattern_{min}$ and $pattern_{m}$.Compare the signal pattern features to identify $pattern_{\mathrm{match}}$.

On the other hand, Kullback–Leibler (KL) Divergence is calculated from minority to majority classes. With this approach, we measure the divergence between the probability distribution represented by minority and majority classes, normalizing the data to represent probability distributions. The KL Divergence applied is expressed in Equation (6),

(6) $$D_{\mathrm{KL}}\left(P\|Q\right)=\sum P\left(i\right)\log\left(\frac{Q(i)}{P(i)}\right),$$

where P(i) represents the probability of observation *i* in $pattern_{min}$ and Q(i) represents the probability of observation *i* in candidate match $pattern_{m}$. After calculating and plotting all the statistical values, we observe the trend of the signals in $pattern_{min}$ and locate similarity in the rest of $pattern_{m}$ from all other rooms to find $pattern_{match}$. We apply both statistical measures to partial and full matching variations of relabeling forming four candidate matches.

To proceed with relabeling, train data $d_{train}$, $pattern_{min}$ for the location with low sample and identified $pattern_{match}$ are required. The expected output is the augmented train data $d_{train,new}$ with concatenated original train data $d_{train}$ and relabeled data $d_{relabeled}$. Listed from one to five are the sequential steps followed to execute the relabeling process. See Algorithm 2.

Nursing homes frequently feature an evenly distributed space across rooms. Given the environment’s influence on signal behavior, the relabeling method is specifically designed for floors where the rooms share the same geometric layout. The relabeling method is exclusively applied to patient rooms in the original floor plan based on matching as depicted in Figure 4. In this study, relabeling is not applied for signals from beacons positioned in areas with diverse dimensions and layouts, such as open spaces in the cafeteria. It is crucial that the beacon devices are installed in fixed positions. Moreover, as the proposed approach specifically filters data to the six surrounding beacons, Standard Deviation and KL Divergence were chosen to measure signal patterns as they represent the variability of the targeted signals. To apply relabeling to other sensors, the representation of signal patterns can be updated using other statistical measures that better suit the behavior of the data being analyzed.  
**Algorithm 2:** Algorithm for relabeling the location of matching class**Input:**$d_{\mathrm{train}}\leftarrow$ train data$pattern_{match}$, $pattern_{min}$**Output:**$d_{\mathrm{train},\;\mathrm{new}}\leftarrow$$d_{\mathrm{train}},\;d_{\mathrm{relabeled}}$Create a dataframe for the relabeled data $d_{\mathrm{relabeled}}$.Populate $d_{\mathrm{relabeled}}$ with values from $pattern_{match}$ following the same columns representing RSSI values of the six beacons.Add remaining columns from $d_{\mathrm{train}}$ in $d_{\mathrm{relabeled}}$ for beacons not included in $pattern_{match}$. Fill with zero values.For relabeling, assign the location of the minority class to the labels for $d_{\mathrm{relabeled}}$.To oversample, combine the augmented data $d_{\mathrm{relabeled}}$ to the original train split  $d_{\mathrm{train}}$ in a new dataframe $d_{\mathrm{train},\mathrm{new}}$.

### 3.4. Indoor Localization

In this paper, indoor localization is approached as a recognition problem using machine learning [72] to identify semantic room locations. This approach is designed to enhance caregiving records by emphasizing the functional nature of spaces within the caregiving environment rather than their physical coordinates. For room estimation considering attenuation, RSSI values from all rooms are considered in feature extraction. Discrepancies between the timestamps of location labels and RSSI data are resolved by synchronizing data. To proceed with indoor localization, statistical and temporal features are derived from the RSSI matrix. The Random Forest algorithm is employed for identifying locations as this classifier demonstrates effectiveness in handling imbalanced data [73] and preferred in indoor positioning [74,75,76]. As we deal with a similar scenario of data imbalance between locations, we opt to adopt this model to focus on relabeling.

Five statistical features are extracted, specifically mean, standard deviation, minimum, maximum, and RSSI count. Integrating the quantity of detected signals per device into the feature set improves the model’s effectiveness, as observed in prior work. Time-based attributes, specifically hour, minute, and microsecond, are extracted. A non-overlapping time window of 45 s is implemented. The train and test data division is date-based, adhering to train-test ratios recommended by empirical studies. The training set comprises 3.5 days of data, and the remaining 1.5 days are intended for test data. Both training and test sets reflect the caregiving routine in the facility, where nursing staff frequently visited the common area compared to the patient rooms.

To assess the effectiveness of relabeling, the indoor localization performance of baseline train data is compared to the performance of data augmentation. In addition, various data augmentation methods must be applied to the minority class. For cross-validation, the relabeling approach is applied to different rooms and compared with other methods. The next chapter covers the evaluation of real-world data collected in the nursing home.

## 4. Data Collection and Evaluation

The majority of the caregiving activities are location dependent; hence, monitoring the rooms visited by nursing staff is necessary for an estimation of the delivered workload and assistance given to elderly individuals on the floor. This information is relevant in automatic care recording, necessitating inputted FonLog data. The nursing care tasks executed by the caregiver fall under the category of patient care, medical assistance, documentation, cleaning, and organization. As the movement of the caregiver is dependent on nursing activities, we elaborate the details on caregiving to record the workload of staff performed in respective locations. Patient care covers bathing, excretion assistance, daytime support, meal preparation, and recreation. Medical assistance encompasses checking patient vitals, administering treatment, and overseeing rehabilitation. Cleaning and organization tasks include changing linens and handling laundry. Care record falls under documentation.

### 4.1. Data Collection

**Figure 6:**
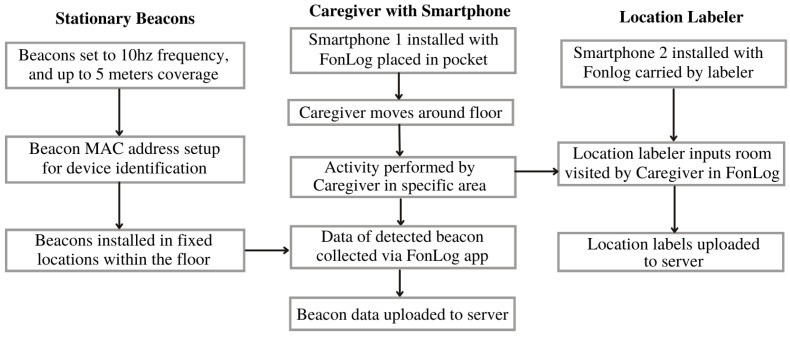
Overview of data collection approach in nursing facility.

The data collection approach implemented in the nursing facility is depicted in Figure 6. Pre-deployment testing was conducted with the caregiver to ensure the detection of beacons, connection to the server, and correct reading of both location labels and BLE signals. Furthermore, initial data gathering with the placement of beacons was conducted to make adjustments accordingly following the nursing home’s protocol. Initially, user feedback was collected via discussion with the participating staff and user study during the pre-deployment setup. A location labeler was employed to address the staff’s challenges in recording locations while carrying out their tasks.

Final data collection was performed on the fifth floor of a nursing care facility over a period of five days, from morning to afternoon of April 10 to 14, capturing the locations a caregiver visited during their daily routine. Daily, about seven hours of data collection was performed: three hours in the morning and four hours in the afternoon. The schedule followed the caregiving routine in the facility. In adherence, the placement of beacons outside patient rooms was decided to protect privacy, and data collection was executed following the facility’s protocol and proposed schedule.

**Figure 7:**
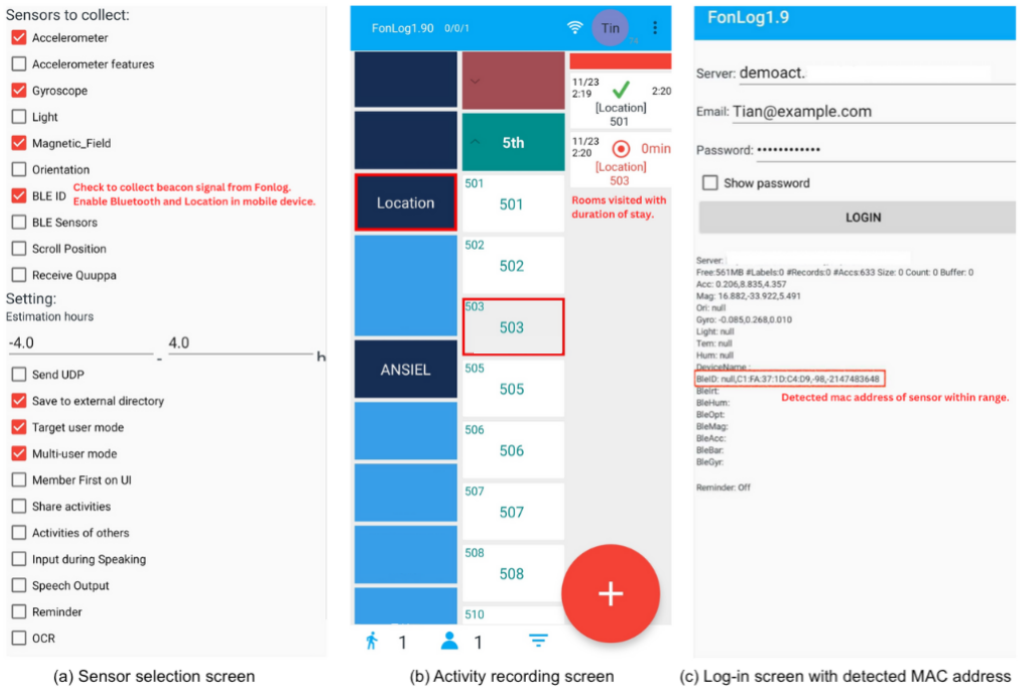
User interface of the FonLog application. (**a**) Sensor selection screen where BLE ID is enabled. (**b**) Activity recording screen showing respective rooms within the floor. (**c**) Log-in interface reflecting detected mac address of BLE device.

FonLog, a smartphone application in Figure 7, was installed and used as a data collection tool to log location labels and as a receiver of RSSI signals [39]. In this study, FonLog was explicitly customized to record activities performed on the respective floor of the nursing care facility with strategically positioned beacons.

Two separate smartphones installed with FonLog were set up for data gathering, one for the nursing staff with enabled BLE ID and another for the location labeler. Prior to deployment, the location and Bluetooth settings of the smartphone were set, and beacon detection was verified on the login interface, where the MAC (Media Access Control) addresses are displayed. The nursing staff was requested to bring one smartphone as they performed caregiving across various rooms on the floor. To avoid disturbance during tasks and maintain the quality of nurse care, the device was placed in the front pocket of the uniform.

A common challenge of on-site data collection is a mismatch between beacon data and the corresponding location label, which occurs due to the delay in activity recording. Caregivers usually log the activity after completing various tasks, resulting in discrepancies in timestamps during pre-processing. To mitigate this problem, the data collection process included an observer closely monitoring the caregiver’s daily routine. The labeler assigned was positioned in the hallway and recorded locations on the second smartphone, with Fonlog choosing from the list of rooms in the app. Both the carer and labeler were each assigned a unique user ID. Similarly, each patient room and area within the floor was allocated a unique customer ID. All beacon data and labels were temporarily stored in the local storage of each smartphone and uploaded to the same server once internet connection was established. In this study, the data collection involved participating nurses of average height. Any significant height differences should be noted, and the impact on RSSI values should be observed in the case of multiple participants.

**Figure 8:**
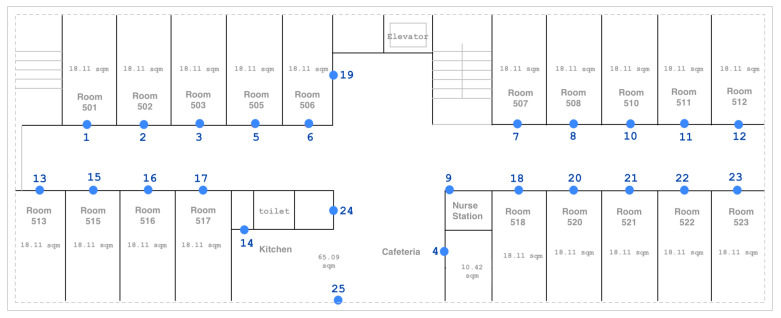
Layout of the facility showing the placement of installed beacons at each location.

Beacon data recorded from Fonlog contain user_id, timestamp, MAC address, and RSSI, while location data by labeler consists of user_id, activity performed, start and stop time, customer_id, and specified location. In our initial approach, we aligned the timezones of both the location and beacon data, and the timestamps were subsequently reformatted. Duplicates and rows with null values were removed, and entries lacking in start-time and stop-time with undefined duration were filtered from location labels. Figure 8 depicts the map of the target floor in the nursing facility with installed beacons.

Before deployment, beacon frequency and height placement were decided based on experiments performed in the lab and hallway of the university, reflecting the facility’s layout. In total, 25 stationary beacons indicated by blue points on the map were installed 2 m from the ground, strategically positioned outside 19 elderly rooms, and in common areas to cover the usual route of the nursing staff. All beacons were configured to a frequency of 10 Hz, where the actual data showed RSSI detection ranging from 3 to 5 Hz.

In medical and elderly care facilities, there are substantial limitations on where BLE beacons can be placed. In scenarios where users carry the beacon tag, scanners are strategically positioned in corners and specific areas, taking into account the geometry of the location [9]. This setup allows for position calculation using methods that do not rely on machine learning. For this research, the placement of beacons was determined in agreement with the nursing home to effectively track the movement of caregivers on the respective floor. Specifically, beacons were mounted at an appropriate height on the doors outside each patient room. This approach was adopted to respect privacy and to minimize disturbance to the elderly during the installation and maintenance of the devices. In contrast to other studies [9], beacons were placed in stationary positions as emitters while Fonlog installed in the caregiver’s smartphone served as the scanner.

The transmission power of beacons varies depending on the calibration of the RSSI at a distance of one meter. Beacons configured for broader coverage have higher transmission power. According to the specification sheet of the BLE device used, an RSSI detection range of 100 m corresponds to a transmission power of +4 dBm. All beacons were set to a detection range of zero to five meters coverage with a transmission power of up to −30 dBm (decibel-milliwatts).

**Figure 9:**
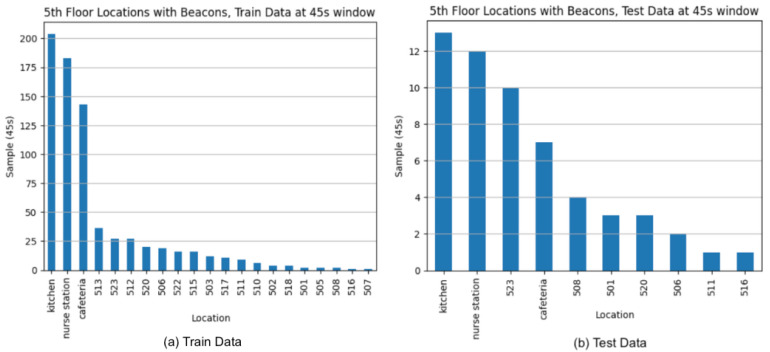
Baseline beacon data with location labels segmented for feature extraction. (**a**) Training split, 45 s window (**b**) Test split, 45 s window.

Figure 9 displays the preprocessed beacon data with location labels used in the baseline of the indoor positioning model.

From the data, we can see an overview of the caregiving routine, which involves more extended activities in the kitchen, nurse station, and cafeteria, where meal assistance and nurse care recording are performed. We can also identify rooms with more extended data, suggesting longer patient assistance is delivered in the respective elderly room. The same test data from the figure are retained to evaluate and compare the performance of different data augmentation approaches in Section 4.3.

### 4.2. Performance Evaluation

As this study aims to address data imbalance by relabeling the approach, a comparative analysis of signal patterns and an assessment of the efficacy of partial and full room matching are conducted. Furthermore, we evaluate the performance of relabeling against other data augmentation methods. The test data cover 1.5 days, while the training data encompass 3.5 days, both segmented into 45 s windows without overlapping.

The target use case for evaluation in this study is centered on enhancing the performance of indoor localization through the meticulous comparison of signal patterns by specific features. By implementing partial and full matching of rooms, we aim to refine the relabeling process and ensure the most accurate representation of location data. This comparison also extends to assessing the efficacy of our proposed methods against other data augmentation approaches, thus providing a comprehensive analysis of performance improvements in indoor localization systems.

To evaluate the impact of data augmentation to indoor positioning performance, F1-score, Precision and Recall are measured and obtained, respectively, using the following Equations in (7)–(9), where TP is the True Positive, TN is the True Negative, FP is the False Positive, and FN the False Negative values.

(7) $$F1-score=\frac{TP}{TP+0.5(FP+FN)},$$

(8) $$Precision=\frac{TP}{\left(TP+FP\right)},$$

(9) $$Recall=\frac{TP}{\left(TP+FN\right)}.$$

**Figure 10:**
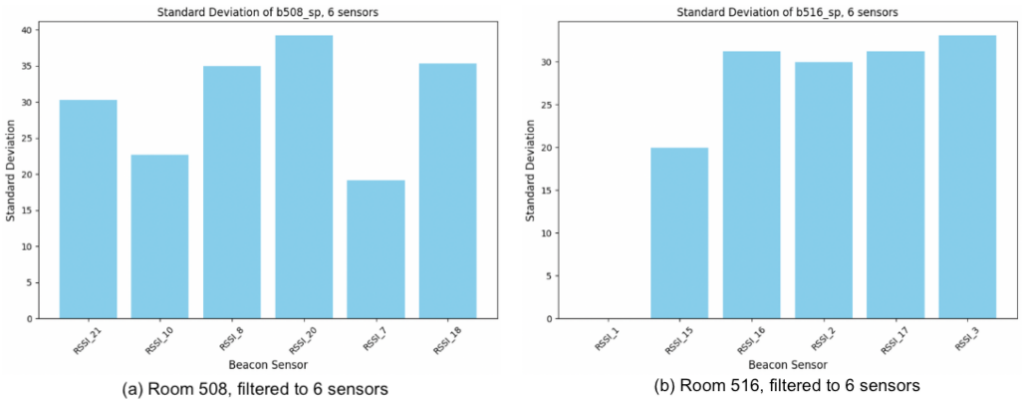
Standard deviation of selected sensors in target minority class rooms. (**a**) Room 508, filtered to 6 sensors (**b**) Room 516, filtered to 6 sensors.

We assess the efficacy of applying relabeling to underrepresented locations within the training dataset from the collected data. For this evaluation, reviewing the beacon data for each location in the training set, Rooms 516 and 507 are identified as minority classes. Filtering the device of all the rooms to six beacons following Equation (4) and Figure 4, we proceed with calculating the signal pattern feature. Figure 10 illustrates the resulting standard deviation of each of six beacons in $pattern_{min}$ corresponding to the minority class rooms.

We then find the match to the minority class by comparing the signal pattern feature of other rooms using standard deviation and KL divergence. Based on the floor layout, corner rooms with only less than six beacons are reserved as candidate matches in partial matching. In comparison, middle rooms with complete six beacons mounted in surrounding locations are considered in full matching. This process allows for two types of relabeling: full and partial matching, by correlating beacons across different classes.

The integration of two variations of matching and two statistical measures for signal pattern features results in four potential scenarios for candidate matching in the relabeling process. To identify the best combination for relabeling, we implement data augmentation to all four use-case scenarios and compare it with other oversampling methods. From this approach, we aim to identify the statistical measure that better represents signal patterns from six beacons, and by comparing full and partial matching, we determine the locations to consider for relabeling.

**Figure 11:**
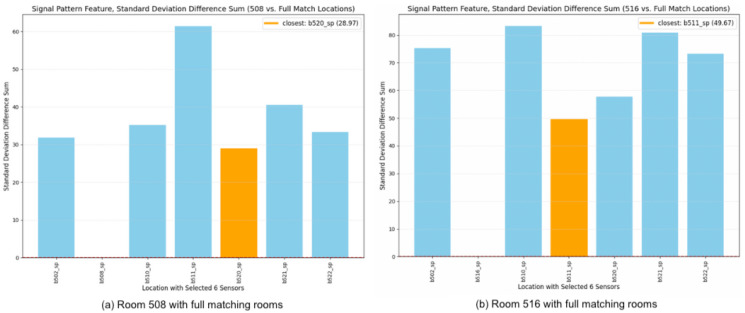
Full matching with signal pattern feature based on standard deviation. (**a**) Room 508 vs. other locations. (**b**) Room 516 vs. other locations.

In the case of full matching using signal patterns determined by standard deviation, as depicted in Figure 11, Room 520 emerges as the closest match to Room 508, and Room 511 is identified as the nearest match to Room 516.

**Figure 12:**
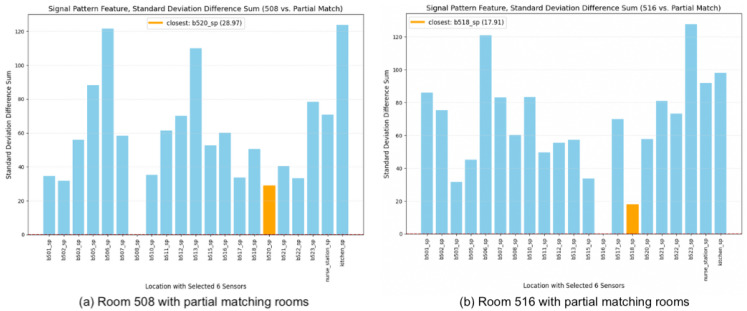
Partial matching with signal pattern feature based on standard deviation. (**a**) Room 508 vs. partial matching locations. (**b**) Room 516 vs. partial matching locations.

Figure 12 reveals that, under partial matching with signal pattern analysis based on standard deviation, Room 520 remains the closest match to Room 508, while Room 518 is identified as the nearest match to Room 516.

**Figure 13:**
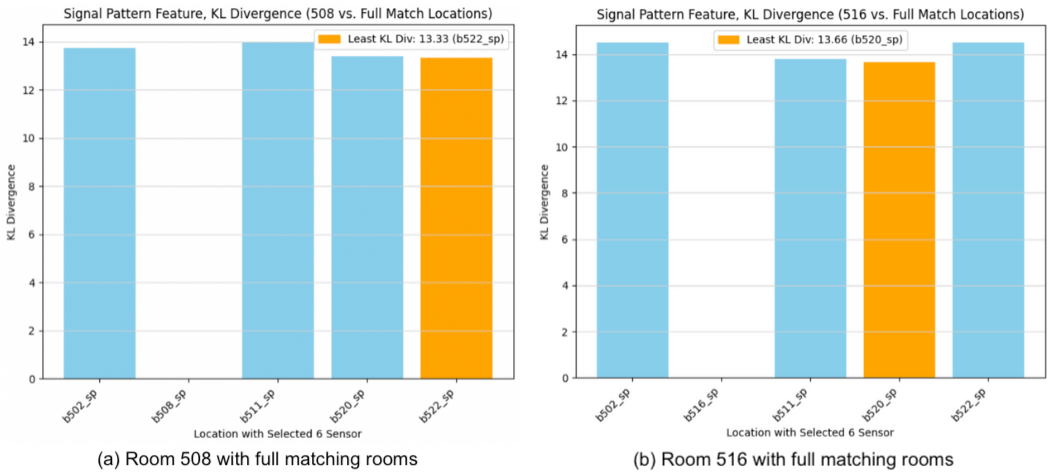
Full matching with signal pattern feature based on KL divergence. (**a**) Room 508 vs. other locations. (**b**) Room 516 vs. other locations.

Continuing with the assessment of the signal pattern using KL divergence, Figure 13 shows the result of full matching with KL divergence where we identify 522 as the majority class nearest match to 508. At the same time, 520 is the match for 516.

**Figure 14:**
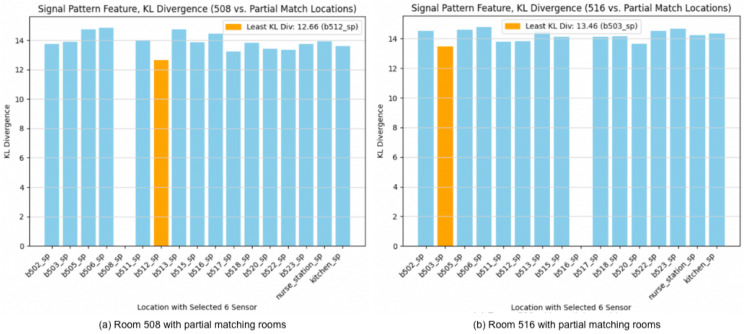
Signal pattern feature based on KL divergence with partial matching. (**a**) Room 508 vs. partial matching locations. (**b**) Room 516 vs. partial matching locations.

Lastly, partial matching based on KL divergence results in 512 as the nearest match of 508, while 503 is identified as the top match for 516 as reflected in Figure 14.

With the matching location identified for each minority class in all four cases, we proceed with relabeling the identified match for each minority location. We concatenate the augmented data to the original training oversampling Rooms 508 and 516. Indoor localization with the Random Forest model is executed using the new training data. Similarly, we apply data augmentation using Random Sampling, ADASYN, and SMOTE to both Room 508 and 516, generating the same length of augmented data as that of the relabeled matches for comparison.

### 4.3. Results

In our evaluation of indoor localization, we assessed the effectiveness of various combinations of relabeling approaches and statistical measures applied to signal patterns. This assessment aimed to determine the specific combination that yields the most optimal performance in terms of minority class localization.

Comparing the performance of relabeling using full matching based on standard deviation in Table 2, only the proposed relabeling method classified Room 516 after oversampling. Relabeling achieved the highest target class F1-score for both 508 and 516. In the overall model F1-score, relabeling improved model performance by 6%. ADASYN achieved the highest value, followed by the proposed method.

The performance of relabeling with partial matching based on standard deviation is summarized in Table 3. In target class F1-score, no oversampling method was able to classify Room 516. At the same time, all data augmentation approaches improved the performance for 508, with SMOTE as the highest, followed by relabeling and ADASYN. Applying Random Sampling to the minority class resulted in a lower F1-score. Regarding the overall weighted F1-score, only ADASYN and SMOTE improved the model by 2 to 3%. As for the relabeling approach, the observed decrease in performance can be attributed to incomplete beacon data resulting from partial matching and the possibility of overlapping classes.

On the other hand, performance comparison with full matching based on the KL divergence is depicted in Table 4. With this relabeling approach, only the proposed method was able to classify Room 516 after performing data augmentation. Relabeling achieved the highest target class F1-score for both Room 508 and 516. In the overall model F1-score, relabeling improved the model performance by 8% and achieved the highest result.

Lastly, Table 5 summarizes indoor positioning comparison with partial matching based on KL divergence. Similarly, in target class F1-score, no oversampling method was able to classify Room 516, while relabeling achieved the highest performance for Room 508 at 73%, which is 16% to 29% higher than that of other oversampling methods. In the overall model F1-score, only relabeling resulted in an improved model performance at 62%.

## 5. Discussion

In this section, we outline the findings of our study, building upon the methodologies and approaches discussed previously. We highlight the contribution to the field of indoor localization, particularly in the context of nursing care facilities.

**Figure 15:**
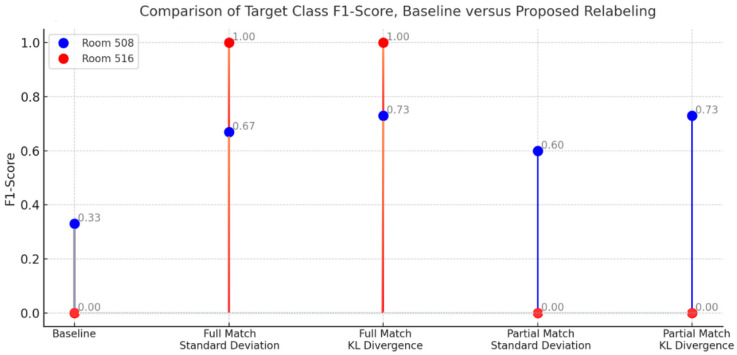
Comparison of Target Class F1-Score, Baseline versus Proposed Relabeling.

To investigate the impact of relabeling on indoor localization in nursing facilities, we illustrate the performance of the proposed method in Figure 15. In response to the first research question on identifying matching signal patterns between different locations in the facility, we calculate signal pattern features using standard deviation to compare per beacon variability and KL divergence to compare signal distributions between rooms. To better understand signal patterns, we filter the analysis of beacons to six surrounding devices in the neighboring rooms.

**Figure 16:**
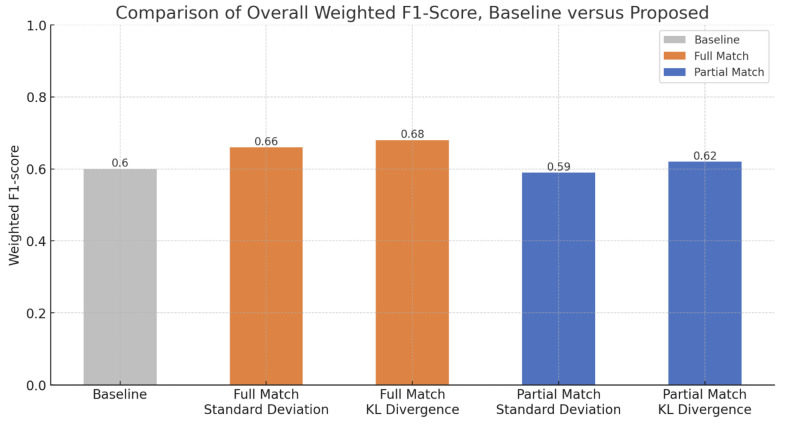
Comparison of Overall Weighted F1-Score, Baseline versus Proposed Relabeling.

In response to the second research question on how we use samples from other beacons to augment location with fewer data, we apply relabeling to the resulting match from the comparison of signal patterns between rooms. We implement two variations to relabeling based on the completeness of six beacons filtered around the surrounding location. We summarize the resulting performance of applying relabeling to the stationary beacons installed in the nursing facility in Figure 15 and Figure 16.

Between the variations of relabeling, full matching consistently achieves better performance compared to baseline and partial matching in terms of the target class F1-score. As reflected in Figure 15, Room 516 is only classified after employing relabeling with full matching. Matching based on signal pattern features referenced from KL divergence results in a better F1-score than standard deviation. Overall, the proposed method improves the target class F1-score compared to the baseline by 27% to 40%.

By comparing the overall F1-score performance in Figure 16, we determined that full matching achieves better performance than baseline with an increase of 6% to 8%. Moreover, full matching outperformes partial matching by 6% to 7%. For both full and partial matching, KL divergence results in a better F1-score than standard deviation. Overall, the proposed method improves model performance compared to the baseline, except in the case of partial matching with standard deviation. In general, increasing the training data with relabeling based on signal pattern results in improved localization.

**Figure 17:**
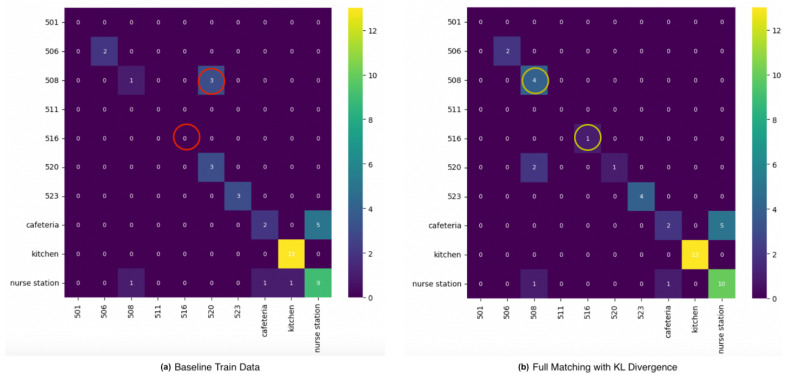
Comparison of indoor positioning with baseline data. (**a**) Confusion matrix, original data. (**b**) Confusion matrix, with relabeled data.

Choosing the best combination of relabeling from the matching variation and signal pattern feature, we compare full matching based on KL divergence with the baseline confusion matrix as depicted in Figure 17. For target class F1-scores, relabeling improves the performance of both Room 508 and 516. All samples of 508 and 516 are correctly localized with relabeling. In the overall model F1-score, relabeling improves performance by 8%. On the other hand, a trade-off of the proposed method is potential class overlap, especially from the majority class used in the matching, which can be observed in Room 520 in the confusion matrix affecting the recognition of the room.

For cross-validation, we test the proposed method by applying data augmentation on other rooms using the same test data. In the original train data, we reduce Rooms 520 and 523 samples and then re-evaluate the performance for baseline before applying augmentation. Table 6 summarizes the result after oversampling Room 520. Relabeling improves both overall and target F1-score of baseline after applying data augmentation and increasing the samples by 96.7%.

For Room 523, the proposed relabeling achieves both the highest overall and target F1-score after applying data augmentation to the room, increasing the samples five-fold the original size as depicted in Table 7.

Given the varying quantities of samples added through oversampling, evaluating and contrasting the augmented data produced by each technique is crucial, as they each function distinctly.

Presently, we remain positive that the application of the proposed method to other rooms relevant insights into the current effectiveness of relabeling. Between the variations in relabeling, full matching effectively utilizes all sensor data from other beacons. Conversely, while partial matching demonstrates some improvement in localization accuracy, its reduced retention of beacon data from other rooms can lead to diminished performance. Overall, as relabeling is based on leveraging samples from other beacons, the proposed data augmentation approach relies on the number of samples in the majority class. In line with other oversampling techniques, the volume of data gathered within the facility for training significantly influences the outcome of data augmentation and the performance of indoor localization.

## 6. Conclusions

In this paper, we proposed a relabeling method for data augmentation towards indoor localization in nursing care facilities. Our proposed method addresses the challenge of data imbalance due to unequal representation of locations with low samples in minority classes. By filtering BLE devices to surrounding beacons, we calculated the signal pattern feature of minority and majority classes and identified matching rooms for relabeling. By applying a relabeling approach to identified locations matching the signal patterns of the minority class, indoor localization improved both in the target class and overall model performance. Moreover, we presented a comparative performance of indoor localization using different augmentation techniques and confirmed that our proposed method achieves better indoor localization in nursing homes.

The main contribution of this work is utilizing real data from other beacons referenced from the majority class to augment locations with fewer samples. With our proposed full and partial matching based on calculated signal pattern features, we showed the advantage of having a flexible method where the statistical measure can be varied in accordance with the targeted analyzed data. Moreover, our method displayed model generalization leveraging majority class data. On the other hand, dependence on the number of samples of majority classes should be taken into account when applying relabeling.

We could discern similarities between different rooms by calculating signal pattern features. This enabled us to effectively augment locations with limited data samples by repurposing data from matching rooms. By measuring both KL divergence and standard deviation across classes, we mapped the signal patterns among various locations. The approach based on KL divergence consistently outperforms baseline data and other methods, specifically Random Sampling, SMOTE, and ADASYN. However, it is important to acknowledge the potential for class overlap with the matching class. The augmented data should be carefully examined.

The reliance of the proposed method on the volume of majority class samples and the potential for class overlap necessitates careful application and consideration. To this end, our future work will focus on expanding data collection to enable more robust evaluations. Broadening the scope of data collection to various nursing homes with different layouts is a key component to validate the effectiveness of relabeling in diverse settings. Furthermore, locations within common areas not following specific layouts will be further examined. We aim to refine our relabeling approach to encompass areas within these common spaces with varying geometries and dimensions, such as cafeterias, where nursing activities tend to occur over extended periods. Applying the relabeling approach to other use-case scenarios using different sensors, such as an accelerometer and gyroscope, will be analyzed to understand signal patterns for matching. The aim is to apply the proposed relabeling to other sensor data to assess the performance in improving models after data augmentation.

Currently, the analysis is performed in the offline phase using real-world data. Expanding the system to a more dynamic real-time relabeling and indoor positioning for nursing homes is suggested to adapt to immediate changes in the caregiving environment. Furthermore, elaborating analysis of signal patterns by incorporating additional statistical analysis techniques is suggested to represent signal pattern features of different devices aside from BLE beacons. A hybrid augmentation approach by combining the proposed oversampling method with SMOTE or ADASYN is suggested to resolve the limitations of respective oversampling techniques and further improve indoor localization. Algorithm optimization should be explored to enhance the accuracy and robustness of indoor localization. We plan to investigate the use of additional machine learning algorithms and the implementation of filtering techniques to improve signal quality, potentially increasing the precision of our localization system. On the other hand, the proposed relabeling maintains its effectiveness even with filtered or high-quality signal inputs.

Lastly, as we continue to improve the FonLog application and indoor positioning system, we will integrate user feedback collection via an in-app survey to incorporate suggestions in the system design cycle. This is to ensure adherence to the protocol of the partner facility and adapt to user experience with practical challenges faced in real-world scenarios.

## Figures and Tables

**Table 1:** Collected raw beacon data stored in the server.

User_id	Timestamp	Mac Address	RSSI
90	2023-04-10 10:22:55.589+0900	FD:07:0E:D5:28:AE	−75
90	2023-04-10 10:22:55.599+0900	D2:1C:25:72:FB:E3	−62

**Table 2:** Performance of Relabeling with Standard Deviation, Full Matching versus other Sampling.

Oversampling Approach	Target Class Precision	Target Class Recall	Target Class F1-Score	Overall Model Weighted F1-Score
Baseline *	Room 508 = 0.50	Room 508 = 0.25	Room 508 = 0.33	0.60
	Room 516 = 0.00	Room 516 = 0.00	Room 516 = 0.00	
Random Sampling	Room 508 = 0.67	Room 508 = 0.50	Room 508 = 0.57	0.64
	Room 516 = 0.00	Room 516 = 0.00	Room 516 = 0.00	
SMOTE	Room 508 = 1.00	Room 508 = 0.25	Room 508 = 0.40	0.63
	Room 516 = 0.00	Room 516 = 0.00	Room 516 = 0.00	
ADASYN	Room 508 = 0.50	Room 508 = 0.50	Room 508 = 0.50	0.69
	Room 516 = 0.00	Room 516 = 0.00	Room 516 = 0.00	
Proposed Relabeling	Room 508 = 0.57	Room 508 = 1.00	Room 508 = 0.73	0.66
	Room 516 = 1.00	Room 516 = 1.00	Room 516 = 1.00	

* Original train data with no augmentation applied.

**Table 3:** Performance of Relabeling with Standard Deviation, Partial Matching versus other Sampling.

Oversampling Approach	Target Class Precision	Target Class Recall	Target Class F1-Score	Overall Model Weighted F1-Score
Baseline *	Room 508 = 0.50	Room 508 = 0.25	Room 508 = 0.33	0.60
	Room 516 = 0.00	Room 516 = 0.00	Room 516 = 0.00	
Random Sampling	Room 508 = 0.67	Room 508 = 0.50	Room 508 = 0.57	0.60
	Room 516 = 0.00	Room 516 = 0.00	Room 516 = 0.00	
SMOTE	Room 508 = 1.00	Room 508 = 0.50	Room 508 = 0.67	0.63
	Room 516 = 0.00	Room 516 = 0.00	Room 516 = 0.00	
ADASYN	Room 508 = 0.50	Room 508 = 0.75	Room 508 = 0.60	0.62
	Room 516 = 0.00	Room 516 = 0.00	Room 516 = 0.00	
Proposed Relabeling	Room 508 = 0.50	Room 508 = 0.75	Room 508 = 0.60	0.59
	Room 516 = 0.00	Room 516 = 0.00	Room 516 = 0.00	

* Original train data with no augmentation applied.

**Table 4:** Performance of Relabeling with KL Divergence, Full Matching versus other Oversampling.

Oversampling Approach	Target Class Precision	Target Class Recall	Target Class F1-Score	Overall Model Weighted F1-Score
Baseline *	Room 508 = 0.50	Room 508 = 0.25	Room 508 = 0.33	0.60
	Room 516 = 0.00	Room 516 = 0.00	Room 516 = 0.00	
Random Sampling	Room 508 = 0.67	Room 508 = 0.50	Room 508 = 0.57	0.66
	Room 516 = 0.00	Room 516 = 0.00	Room 516 = 0.00	
SMOTE	Room 508 = 0.67	Room 508 = 0.50	Room 508 = 0.57	0.66
	Room 516 = 0.00	Room 516 = 0.00	Room 516 = 0.00	
ADASYN	Room 508 = 0.60	Room 508 = 0.75	Room 508 = 0.67	0.66
	Room 516 = 0.00	Room 516 = 0.00	Room 516 = 0.00	
Proposed Relabeling	Room 508 = 0.57	Room 508 = 1.00	Room 508 = 0.73	0.68
	Room 516 = 1.00	Room 516 = 1.00	Room 516 = 1.00	

* Original train data with no augmentation applied.

**Table 5:** Performance of Relabeling with KL Divergence, Partial Matching versus other Oversampling.

Oversampling Approach	Target Class Precision	Target Class Recall	Target Class F1-Score	Overall Model Weighted F1-Score
Baseline *	Room 508 = 0.50	Room 508 = 0.25	Room 508 = 0.33	0.60
	Room 516 = 0.00	Room 516 = 0.00	Room 516 = 0.00	
Random Sampling	Room 508 = 0.67	Room 508 = 0.50	Room 508 = 0.57	0.60
	Room 516 = 0.00	Room 516 = 0.00	Room 516 = 0.07	
SMOTE	Room 508 = 0.67	Room 508 = 0.50	Room 508 = 0.57	0.58
	Room 516 = 0.00	Room 516 = 0.00	Room 516 = 0.00	
ADASYN	Room 508 = 0.40	Room 508 = 0.50	Room 508 = 0.44	0.57
	Room 516 = 0.00	Room 516 = 0.00	Room 516 = 0.00	
Proposed Relabeling	Room 508 = 0.57	Room 508 = 1.00	Room 508 = 0.73	0.62
	Room 516 = 0.00	Room 516 = 0.00	Room 516 = 0.00	

* Original train data with no augmentation applied.

**Table 6:** Comparison of Indoor Localization Performance, Oversampling Room 520.

Oversampling Approach	Train Data Room 520	Room 520 F1-Score	Overall Weighted F1-Score
Baseline	1000	0.00	0.56
Random Sampling	1969	0.50	0.67
SMOTE	1969	0.40	0.67
ADASYN	1969	0.86	0.67
Proposed method	1969	0.40	0.63

**Table 7:** Comparison of Indoor Localization Performance, Oversampling Room 523.

Oversampling Approach	Train Data Room 523	Room 523 F1-Score	Overall Weighted F1-Score
Baseline	2178	0.00	0.53
Random Sampling	11,174	0.00	0.53
SMOTE	11,174	0.00	0.53
ADASYN	11,174	0.33	0.59
Proposed method	11,174	0.33	0.61

## Data Availability

The data presented in this study are available on request from the corresponding author.

## Abbreviations

The following abbreviations are used in this manuscript:

ADASYN	Adaptive Synthetic Sampling
AoA	Angle of Arrival
BLE	Bluetooth Low Energy
HAR	Human Activity Recognition
LOS	Line of Sight
IMU	Inertial Measurement Unit
IoT	Internet of Things
IPS	Indoor Positioning System
KL	Kullback–Leibler
MAC	Media Access Control
NLOS	Non-line of Sight
RF	Random Forest
RFID	Radio Frequency Identification
RS	Random Sampling
RSS	Received Signal Strength
RSSI	Received Signal Strength Indicator
SMOTE	Synthetic Minority Oversampling Technique
ToA	Time of Arrival
Wi-Fi	Wireless Fidelity
WLAN	Wireless Local Area Network
